# Quality of end‐of‐life care with non‐malignant liver disease: Analysis of the VOICES National Survey of Bereaved People

**DOI:** 10.1111/liv.15428

**Published:** 2022-09-22

**Authors:** Roberta I. Jordan, Yousuf ElMokhallalati, Lynsey Corless, Michael Bennett

**Affiliations:** ^1^ Academic Unit of Palliative Care, Leeds Institute of Health Sciences University of Leeds Leeds UK; ^2^ Hull York Medical School University of Hull Hull UK

**Keywords:** cirrhosis, palliative care, quality of life

## Abstract

**Background and Aims:**

Patients with liver disease struggle to access palliative care. We aimed to compare carers' perceptions of end‐of‐life care for decedents with non‐malignant liver disease, malignant liver disease and other non‐malignant diseases, and to identify associated factors in non‐malignant liver disease.

**Methods:**

A retrospective analysis of individual‐level data from the National Survey of Bereaved People 2011–2015.

**Results:**

More decedents with non‐malignant liver disease died in hospital than other diseases (76.9% vs. 40.9% vs. 50.2%, *p* < .001), despite 89% wishing to die at home. Fewer decedents received home/hospice specialist palliative care compared with those with malignant liver disease (10.0% vs. 54.6%, *p* < .001). Carers of decedents with non‐malignant liver disease were less likely to rate overall end‐of‐life care quality as outstanding/excellent (29.3% vs. 43.9% vs. 42.3%, *p* < .001). For this group, poorer care was associated with younger (65–74 vs. 18–64 years, OR [odds ratio] 1.39, *p* = .01), more socially deprived decedents (OR .78, *p* = .02), and better care with greater social support (OR 1.82, *p* < .001) and community specialist palliative care involvement (OR 1.80, *p* < .001). There was no association between outstanding/excellent rating and underlying cause of non‐malignant liver disease (alcohol‐related vs. non‐alcohol‐related, *p* = .92) or place of death (hospital vs. non‐hospital, *p* = .476).

**Conclusions:**

End‐of‐life care could be improved by integrating hepatology and community services, particularly specialist palliative care, and advance care planning to facilitate care and death (where desired) at home. However, death in hospital may be appropriate for those with non‐malignant liver disease.

**Abbreviations**
ACP: advance care planning
IMD: Index of Multiple Deprivation
NHS: National Health Service
NIHR: National Institute for Health Research
ONS: Office for National Statistics
OR: odds ratio
RCT: randomised controlled trial
SPC: Specialist Palliative Care
VOICES: Views of Informal Carers – Evaluation of Services

**Lay Summary**

The quality of care at the end of life is poorer for those with non‐cancer liver disease, compared to liver disease caused by cancer and other non‐cancer diseases such as heart failure and kidney failure. This could be improved by ensuring that hospital teams communicate with and provide care alongside community services, such as specialist palliative care. Patients and their families should also be offered chances to discuss and plan their future care so they can avoid any unwanted procedures and spend time where they want.

## 1 INTRODUCTION

There was a 400% increase in liver disease mortality in the UK between 1970 and 2010, with an ongoing rise in the last decade despite public health interventions.^1^, ^2^ Patients with end‐stage liver disease report significant physical, psychological and social burdens, comparable to or even exceeding those of other advanced diseases.^3^, ^4^, ^5^ Concerns have been raised about the quality of end‐of‐life care for patients with non‐malignant liver disease. A national study of over 13 000 patients in England 2013–2015 reported a mean of 35 inpatient hospital days, 3.3 emergency admissions, and total cost of £21 113 per patient in the last year of life.^6^ 75% of deaths occurred in hospital, of which 97% occurred during unplanned hospital admissions. Large US‐based population studies of patients during terminal hospitalisation for end‐stage liver disease or decompensated cirrhosis report that 80% are admitted to ICU, with 56% experiencing mechanical ventilation, 16% haemodialysis initiation and 23% endoscopy.^7^, ^8^ Patients have limited and variable access to specialist palliative care (SPC), with North American population studies reporting rates from 4.5% to 30.3%.^7^, ^8^, ^9^, ^10^, ^11^ When patients are referred to SPC, this largely occurs in the last days/weeks of life with less opportunity for support.^11^, ^12^ Smaller retrospective studies have highlighted low rates of do‐not‐attempt cardiopulmonary resuscitation and advance care planning documentation.^13^, ^14^ Patients report poor information‐sharing about how their disease may progress, symptom management, quality of life and available support services.^4^, ^15^, ^16^, ^17^ Although liver healthcare professionals recognise these needs, they lack confidence and skills discussing them with patients.^17^, ^18^

It is unclear how end‐of‐life care for patients with non‐malignant liver disease compares with those with malignant liver disease, who have similar clinical features, such as decompensated liver failure, and are likely to interact with similar healthcare professionals and services. Furthermore, there is uncertainty as to how this care compares with that of patients with other non‐malignant diseases, which share a similar less predictable disease trajectory than for those with malignant disease.^19^ The National Survey of Bereaved People (VOICES, Views of Informal Carers – Evaluation of Services) was a nationally representative cross‐sectional survey conducted in England annually 2011–2015.^20^ It was commissioned by the Department of Health in 2011 and 2012, and NHS England from 2013, and administered by the Office of National Statistics. Its purpose was to inform and support national policy surrounding end‐of‐life care.^21^, ^22^ It previously reported that carers of decedents where liver disease was mentioned were less likely to rate the overall quality of end‐of‐life care as outstanding, in comparison to those with other diseases.^23^ However, the potential reasons for this are unclear without further analysis. We aimed to compare carers' perceptions of end‐of‐life care for decedents with non‐malignant liver disease, malignant liver disease, and other non‐malignant diseases, and to identify factors associated with the quality of end‐of‐life care for those with non‐malignant liver disease.

## 2 METHODS

### 2.1 Population and data source

We conducted a retrospective analysis of the VOICES survey 2011–2015.^20^ Those who registered the deaths of a stratified sample of around 49 000 adult (≥18 years old) decedents each year were asked about their views on end‐of‐life care, particularly the last 3 months of life, via a postal questionnaire 4–11 months after death. Study variables relevant to end‐of‐life care quality were developed following research with bereaved carers and feedback from survey respondents, and informed by the Department of Health End of Life Care Strategy (2008).^21^, ^24^ Further information on the VOICES methodology is available from the Office for National Statistics (ONS).^24^ VOICES data are available from the UK Data Service to researchers upon application.^20^

### 2.2 Sampling

We acquired individual‐level data from 110 311 completed questionnaires (response rate 45%). We used relevant ICD‐10 codes to identify all decedents with non‐malignant liver disease (hereafter referred to as liver disease: K70.0‐K70.4; K70.9‐K72.1; K72.9‐K73.2; K73.8‐K74.6; K75.0‐K75.4; K75.8‐K77.0; K77.8), malignant liver disease (hereafter referred to as liver cancer: C22.0; C22.1; C22.2; C22.3; C22.4; C22.7; C22.9), and other non‐malignant diseases (hereafter referred to as other disease: codes A00 to R99 “excluding cancer codes C00 to D48.9 and the aforementioned codes for liver disease”).^24^

### 2.3 Study variables

Decedent characteristics included age at death, gender, and index of multiple deprivation (IMD) quintiles (IMD1 = most deprived, IMD5 = least deprived). We reported the proportion of respondents declaring their relationship to the decedent as “wife/partner”, “husband/partner” (Q.54), using this as a proxy measure for social support at home. We only used these two options given the greater confidence that a decedent's wife/husband/partner would be living in the same home as the decedent, than other options such as “son/daughter”, “parent”, “friend” or “neighbour”. We used a proxy measure for SPC at home (defined as proportion reporting yes to receiving help at home from at least one of: a Macmillan nurse, hospice home‐care nurse or specialist; a Marie Curie nurse; and/or Hospice at home, Q.3).^25^ All of these professionals provide SPC in home settings. Macmillan and Marie Curie nurses are clinical nurse specialists in palliative care. If a respondent reported that the decedent had lived or stayed in a hospice during the last 3 months of life (defined as reporting “yes”, Q.29) and/or their response met the definition for SPC at home, we identified that they had received SPC at home or hospice. Other end‐of‐life care variables included proportion of respondents reporting: significant out‐of‐hours urgent care (≥three times, Q.7); good pain relief (defined as proportion reporting “completely, all of the time” or “completely, some of the time”) at home (Q.6), care home (Q.22), hospital (Q.26) or hospice (Q.31); decedent involvement in decision making (defined as proportion reporting “he/she was involved as much as he/she wanted to be”, Q.49); respondent involvement in decision making (defined as proportion reporting “I was involved as much as I wanted to be”, Q.50); help and support for respondent or family by the healthcare team at the actual time of death (defined as proportion reporting “yes, definitely”, Q.47); good coordination between hospital and community services (defined as proportion reporting “yes, definitely”, Q.27); decedent‐expressed preference for place of death (defined as proportion reporting “yes”, Q.42), and those whose expressed preference was home (Q.43); healthcare staff recorded preference for place of death (defined as proportion reporting “yes”, Q.44); actual place of death as hospital (defined as “in a hospital ward”, “in a hospital Accident and Emergency Department”, or “in a hospital Intensive Care Unit”, Q.41), care home (defined as “in a care home”, Q.41), home (defined as “in his/her own home” or “in the home of another family member or friend”, Q.41) or hospice (defined as “in a hospice”, Q.41); thoughts that the decedent had died in the right place (defined as proportion reporting “yes”, Q.46); and rating of overall quality of care in the last 3 months of life as “outstanding” or “excellent” (Q.5).

### 2.4 Statistical analysis

Data were summarised using means and SDs, absolute numbers and weighted percentages or odds ratios (with 95% confidence intervals). The chi‐square test was used to compare the study variables between each group. We conducted logistic regression models using complete case analysis to identify associations between study variables and outstanding or excellent overall quality of care among decedents with liver disease. Independent variables included gender, age, IMD, the respondent's relationship to the decedent, receiving SPC at home and recorded preference for place of death.

The multivariable logistic regression model was constructed using backward stepwise variable elimination procedure. To account for potential collinearity, all variables with a *p* value <.10 univariately were included in the final multivariable model. Study variables with >20% missing data were excluded from the analysis. A significance level of .05 was used. The interaction between independent variables was checked by a multicollinearity test using the variance inflation factor (VIF). IBM SPSS statistics version 24 was used for data management and analysis.

## 3 RESULTS

Our analysis included 1133 decedents with liver disease, 75 203 with other disease, and 395 with liver cancer. Table 1 provides a comparison for the three included groups. All *p* values in these comparisons were significant (<.001).

**TABLE 1:** Decedent and service characteristics by cause of death. Comparisons using chi‐square test. Values are numbers (percentages) of decedents unless stated otherwise. All *p* values in these comparisons were significant (<.001).

	Liver disease (*n* = 1133)	Other disease (*n* = 75 203)	Liver cancer (*n* = 395)
Decedent characteristics			
Age in years; mean (SD)	63.1 (14.0)	84.1 (10.5)	73.1 (11.5)
Male	690 (62.0)	31 344 (43.4)	290 (73.7)
Level of deprivation (IMD quintiles)			
1 and 2 (most deprived)	564 (54.8)	25 847 (37.7)	171 (46.4)
Respondent's relationship to decedent			
Spouse/partner	391 (33.9)	13 760 (20.6)	168 (42.6)
Service and care characteristics			
Received SPC at home or hospice	126 (10.0)	5201 (7.4)	218 (54.6)
Out‐of‐hours urgent care ≥3 visits	200 (25.7)	9243 (27.7)	110 (31.3)
Good pain relief at home	176 (31.2)	9687 (48.0)	156 (51.8)
Good pain relief in care home	48 (66.5)	14 346 (73.9)	39 (71.7)
Good pain relief in hospital	419 (63.6)	21 729 (67.8)	167 (70.0)
Good pain relief in hospice	44 (86.9)	1294 (87.3)	63 (80.5)
Decedent involvement in decision making	555 (79.0)	38 225 (86.1)	268 (81.7)
Respondent involvement in decision making	551 (55.4)	52 150 (77.2)	283 (74.7)
Help and support for respondent or family by the healthcare team at the actual time of death	519 (49.8)	39 687 (59.3)	215 (57.5)
Good coordination between hospital and community services	122 (24.0)	8437 (31.1)	66 (29.8)
Decedent expressed preference for place of death	265 (22.7)	18 718 (25.3)	220 (54.6)
Decedent expressed home as the preferred place of death	237 (88.5)	16 597 (82.2)	186 (82.1)
Healthcare staff recorded preference for place of death	100 (38.2)	7320 (40.2)	141 (63.9)
Actual place of death			
Hospital	836 (76.9)	39 571 (50.2)	148 (40.9)
Care home	69 (4.4)	22 664 (22.5)	41 (9.5)
Home	209 (17.5)	12 231 (21.4)	147 (35.9)
Hospice	19 (1.2)	737 (5.9)	59 (13.7)
Rating overall quality of care in last 3 months of life outstanding or excellent	300 (29.3)	27 753 (42.3)	161 (43.9)

*Note*: All *p* values <.001. All percentages were weighted by sampling weight and non‐response weight.

Abbreviations: IMD, index of multiple deprivation; SPC, specialist palliative care.

Liver disease decedents were significantly younger and had greater levels of social deprivation than the liver cancer and other disease groups. They were more likely to be female with less social support at home than liver cancer decedents. 10% of liver disease decedents received home and hospice SPC compared with 55% of liver cancer decedents and 7% of other disease decedents. They were less likely to have experienced ≥3 out‐of‐hours urgent care visits than the other groups (26% liver disease vs. 31% liver cancer vs. 28% other disease). When compared with those with liver cancer and other disease, liver disease decedents were less likely to have experienced good pain relief at home, care home or hospital settings (more likely in the hospice setting compared with liver cancer); less likely to have experienced good coordination of care between hospital and community services; the decedents and their respondents were less likely to have been involved in decision making; and healthcare staff were less likely to have recorded a preference for place of death. Respondents or family of those with liver disease were less likely to have received help and support by the healthcare team at the actual time of death than for liver cancer and other disease decedents (50% vs. 58% vs. 59% respectively). More liver disease decedents died in hospital than liver cancer and other disease (77% vs. 41% vs. 50% respectively), despite, where expressed (22%), 89% of liver disease decedents wanting to die at home. 75% of respondents for liver disease decedents thought that they had died in the right place. However, this was significantly lower than for liver cancer (82%) and other disease decedents (81%). Carers of liver disease decedents were less likely to rate overall quality of care as outstanding or excellent (29% liver disease vs. 44% liver cancer vs. 42% other disease).

Table 2 summarises the findings of the multivariable logistical regression model. 9.5% of data for overall quality of end‐of‐life care was missing. The analysis showed significantly greater odds of receiving “outstanding” or “excellent” care among liver disease decedents with social support at home (OR 1.39; 95% CI 1.27 to 1.53; *p* = <.001), and receiving SPC at home or hospice (OR 2.44; 95% CI 2.13 to 2.78; *p* = <.001). Healthcare staff recording the preferred place of death (OR 2.44; 95% CI 2.13 to 2.78; *p* = <.001) was also associated with rating the overall care as “outstanding” or “excellent”. Compared with those living in the least deprived quintiles, decedents living in the first and second‐most deprived quintiles had lower odds of receiving “outstanding” or “excellent” care (OR 0.79; 95% CI 0.72 to 0.87, *p* = <.001). Younger decedents (18–64 years) also had lower odds of receiving this level of care compared with those 65–74 years old, as did female decedents in comparison to male. There was no significant difference in the odds of receiving “outstanding” or “excellent” care when comparing decedents aged ≥75 years and those 18–64 years.

**TABLE 2:** Multivariable logistic regression of factors associated with “Outstanding” or “Excellent” Overall quality of care among decedents with liver disease

Effect	OR	95% CI		*p* value
Age of deceased at death (years)				<.001*
65–74 (OR compared to age 18–64)	1.28	1.15	1.42	<.001
75+ (OR compared to age 18–64)	1.09	0.97	1.22	.16
Sex				
Male (OR compared to female)	1.30	1.19	1.43	<.001
Index of multiple deprivation (quintiles)				
IMD 1 and 2 (OR compared to least deprived quintiles IMD 3, 4 and 5)	0.79	0.72	0.87	<.001
Respondent's relationship to decedent				
Spouse/partner (OR compared to other)	1.39	1.27	1.53	<.001
Received SPC at home or hospice				
Yes (OR compared to no)	2.44	2.13	2.78	<.001
Recorded preference for place of death				
Yes (OR compared to no)	2.44	2.13	2.78	<.001

Abbreviations: IMD, index of multiple deprivation; OR, odds ratio; SPC, specialist palliative care.

* *p* value for overall effect.

Pain relief at home and coordination between hospitals and community services were variables removed from the analysis owing to >20% missing data. Furthermore, aetiology of liver disease and place of death had p values of greater than .10 univariately, (*p* = .92 and *p* = .476, respectively) and were also excluded from the analysis. The results show no multicollinearity, since VIF scores were less than 1.5.

## 4 CONCLUSIONS

We aimed to compare carers' perceptions of end‐of‐life care for decedents with liver disease, liver cancer and other disease. This analysis found that those with liver disease had a greater degree of social deprivation, had less involvement and coordination of end‐of‐life care, and were more likely to die in hospital with carers reporting a lower quality of overall end‐of‐life care. Decedents with liver disease received less home and hospice SPC than those with liver cancer. Decedent factors associated with better quality of end‐of‐life care include older age, lower levels of social deprivation, and social support at home. Similarly, associated service factors include SPC at home or hospice, and healthcare staff recorded preferred place of death.

This study builds a more detailed picture of end‐of‐life care comparisons between patients with non‐malignant and malignant liver disease. Peng et al.'s national observational study of all non‐accidental adult deaths from liver disease in England 2001–2014 reported that people who died from liver cancer were less likely to die in hospital than those with alcohol‐related liver disease, but more likely than those with fatty liver disease.^26^ 61% of liver disease decedents from the VOICES study died from alcohol‐related liver disease. This potentially explains the greater proportion dying in hospital than liver cancer. Those with alcohol‐related liver disease have complex psychological and social problems including guilt, issues surrounding abstinence and social isolation, and they struggle to interact with medical services.^4^, ^5^, ^27^, ^28^ They have more portal hypertensive complications as well as all‐cause, cirrhosis‐related and alcohol‐related hospital admissions than for non‐alcohol‐related liver diseases, potentially explained by delays in accessing medical care and ongoing alcohol use.^29^ Reassuringly, cause of liver disease (alcohol‐related vs. non‐alcohol‐related) was not included in the final logistical regression model, as it was eliminated owing to non‐significance, suggesting that carers' perceptions of end‐of‐life care were grossly comparable. Hudson et al. report a lower proportion of patients with hepatocellular carcinoma (HCC) accessing unplanned care in comparison with those with alcohol‐related liver disease, but greater than those with viral liver disease and non‐alcoholic steatohepatitis.^6^ Overall, this study reported better end‐of‐life care outcomes for patients with HCC compared with those with non‐malignant liver diseases including fewer inpatient bed days, unplanned hospital deaths, and lower associated costs. Patients with HCC may receive more information on disease trajectory and prognosis.^4^ They frequently report pain which may prompt palliative care referral.^5^ Several studies report greater odds of access to SPC and hospice care if patients have a diagnosis of HCC compared with those without this diagnosis.^8^, ^9^, ^10^, ^30^ A national US population‐based study comparing deaths from cirrhosis with cancer more broadly reported lower rates of hospice utilisation in cirrhosis.^31^ Our results are consistent with literature highlighting inequitable access to palliative care for those with non‐malignant disease compared with malignant disease.^12^, ^32^, ^33^, ^34^ This reflects concerns such as patient perceptions of palliative care, uncertainty in predicting prognosis, a lack of expertise of palliative care teams in managing non‐malignant conditions, the historical association between palliative care and cancer and a fear of SPC services being overwhelmed by referrals from this population.^34^, ^35^, ^36^

To our knowledge, this is the first study which primarily aims to compare a wide range of end‐of‐life care outcomes for patients with non‐malignant liver disease and other non‐malignant diseases. A UK retrospective cohort study of over 42 000 decedents referred to hospice‐based palliative care reported that non‐cancer liver failure patients were referred a median of 16 days prior to death, compared with 24 days for neurodegenerative diseases (excluding motor neurone disease), 35 days for kidney failure, 40 days for lung disease and 67 days for heart failure.^12^ Conversely, a single‐centre retrospective study from Canada comparing patients with non‐cancer end‐stage liver disease and those with other non‐cancer diseases (majority cardiovascular disease and dementia) reported comparable lengths of stay in an inpatient palliative care unit.^37^ A further single‐centre study from Taiwan found favourable rates of do‐not‐resuscitate orders and hospice utilisation of those with chronic liver disease and cirrhosis compared with other non‐cancer diseases.^38^ However, these studies are limited by small sample sizes and may not be representative of larger populations. We have found a concerning disparity in end‐of‐life care outcomes for liver disease decedents in comparison with decedents with other diseases. Recognised barriers include identification of end‐stage disease, integration of supportive and palliative care, and confidence and skills in discussing prognosis and disease trajectory with patients and their carers.^16^, ^17^, ^18^ However, these barriers are applicable to non‐cancer diseases more generally.^34^, ^35^, ^36^ The disparity identified may represent liver‐specific issues such as the dilemma involved in integrating palliative care alongside consideration for curative liver transplantation, and the social complexity of those with alcohol‐related liver disease and its impact on access to health care. It may also indicate a need for culture change allowing parallel planning of disease modification and palliative care within liver healthcare services.

Our study highlights the potential and specific benefit of community‐based SPC for improving overall end‐of‐life care for patients with liver disease. Systematic reviews assessing the effect of SPC highlight benefits in symptom management, quality of life, reduced hospitalisation and associated costs.^39^, ^40^, ^41^ However, these largely focus on patients with malignant disease. Quinn et al. reported a systematic review evaluating randomised controlled trials (RCTs) of palliative care interventions for patients with non‐malignant disease.^42^ Benefits are consistent with previous systematic reviews, except for quality of life. To date, no RCTs have been conducted evaluating the effects of SPC for patients with liver disease. However, this paper contributes to a growing body of observational studies reporting associations between SPC and better symptom management, reduced hospitalisation, invasive procedures, deaths in hospital and associated costs, increased hospice use and better overall quality of life.^8^, ^10^, ^43^, ^44^, ^45^, ^46^ A feasibility trial of a 6‐month palliative care intervention delivered by a supportive care liver nurse specialist for patients with advanced liver disease was found to be acceptable by patients with statistically significant improvements in quality of life, patient‐reported palliative care outcome scores and electronic records facilitating communication between different settings.^47^ Ufere et al.'s observational study of hospitalised patients receiving palliative care consultations reported similar improvement in symptom improvement and changes in resuscitation preference between those with liver disease and those with cancer.^48^ May et al. highlighted greater reductions in healthcare costs associated with palliative care for those with liver failure, compared with other non‐cancer diagnoses.^49^

Our study found a greater proportion of liver disease decedents dying in hospital compared with decedents with liver cancer and other diseases, despite 89% of those expressing a preference for place of death choosing home. Although hospital deaths possibly reflect more aggressive treatment and healthcare‐associated costs at the end of life, it is important to recognise that this is not always an undesirable outcome for many individuals. It may reflect familiarity with and trust in hospital hepatology teams or the potential for sudden distressing events prompting hospital transfer (e.g. variceal bleeding). Only 22% of decedents with liver disease had actually expressed a preference for place of death. Place of death (hospital vs. other settings) was eliminated from the logistic regression model owing to non‐significance, suggesting carers' perceptions of end‐of‐life care were grossly comparable between hospital and non‐hospital settings. 55% of decedents with liver disease had significant levels of deprivation, which may contribute to unstable home environments and more challenging advance care planning (ACP). However, there may also be a lack of established practice of ACP in hepatology.^14^, ^50^ 75% of respondents felt the liver disease decedents had died in the right place, however, this was significantly lower than for the other decedents. The study highlights a potential wish of certain liver disease decedents (and their carers) to die at home. There is a misconception that sudden events such as catastrophic variceal bleeding must be managed in hospital. Similar bleeding events are planned for and managed in home and hospice settings for patients with other cancers with a high risk of bleeding. Healthcare staff recorded preference for place of death, a potential proxy for ACP, was associated with outstanding/excellent quality of end‐of‐life care for liver disease decedents. Despite a lack of high‐quality RCTs and liver disease‐specific data, systematic reviews evaluating ACP found potential benefits for reduced hospitalisation and aggressive interventions at the end of life, as well as involvement with hospice and palliative care.^51^, ^52^, ^53^ We should be open to discussions with patients about their wishes including their place of care and death. Where possible, this will allow greater planning for deaths in various settings, including hospital where preferred.

A key strength of this work is the large dataset across 5 years with a modest questionnaire response rate of 45% (high for this type of survey methodology). This allows for reasonable national generalisability and good statistical power. This is the first study reporting on comparisons of decedent‐ and service‐level end‐of‐life care outcomes between liver disease, liver cancer and other disease. It highlights discrepancies for end‐of‐life care for those with liver disease and potential factors which may improve this care. It also highlights disparities in end‐of‐life care associated with difference in demographics, for example, age, level of deprivation, building upon existing literature and reports.^12^, ^54^ We focused on questions relating to the last 3 months of life from the survey reflecting the importance of early palliative care and the significant burden of symptoms found in patients with advanced liver disease, not just in the last days of life.

Limitations include the respondents being relatives/friends of the decedents rather than the decedents themselves, a potential lack of clarity as to who completed the postal survey, potential misinterpretations of the survey and the potential for recall bias (mitigated by the timing of the questionnaires). This may have led to an inaccurate reflection of the decedents' experiences. Coding errors and inaccurate death certification may have led to incorrectly assigned diagnostic groups. Furthermore, small sample sizes, particularly for liver cancer, and missing data, restricted a more extended analysis including comparisons between the 5 years of data collection and use of ethnicity data. There were no relevant questions relating to social support at home and access to SPC in any setting and therefore proxy measures were designed to capture these variables, potentially leading to inaccuracies. The VOICES survey has been criticised by some respondents as being limited in its ability to capture their true experience owing to its tick‐box design and is limited in its range of questions regarding symptom management, largely focusing on pain. It is well recognised that patients with liver disease and other diseases experience a broad range of symptoms.^3^ Ultimately the dataset was collected 5–10 years ago and although there remains a recognised need for improvement in end‐of‐life care for liver disease, it is possible that the findings are not up‐to‐date with current practice.

The study reflects existing concerns about end‐of‐life care for those with liver disease. It also suggests poorer care in comparison with those with liver cancer and other diseases in the UK. It highlights potential factors which could improve care such as access to community SPC. It recognises the potential desire and benefit of ACP for patients with liver disease. Further research is needed to better understand the attitudes of patients with liver disease towards ACP and palliative care. This would help in the design of an intervention supporting integration of relevant services and better access to community support. Lessons can be learnt from the integration of palliative care with disease management of other non‐malignant diseases such as heart failure, particularly how barriers to end‐of‐life care have been overcome. However, any intervention must also consider factors specific to patients with liver disease including the prospect of liver transplantation and the potential complex social circumstances of those with alcohol‐related liver disease which may impact on access to end‐of‐life care.

## AUTHOR CONTRIBUTIONS

RIJ and YE conceived the initial study concept. RIJ, YE, LC and MB wrote the study protocol. YE attained and analysed the study data, with verification by RIJ, LC and MB. RIJ, YE, LC and MB drafted and revised the manuscript. All authors confirm they had full access to all the data in the study and accept responsibility to submit it for publication.

## FUNDING INFORMATION

This research was supported by the National Institute for Health Research (NIHR) infrastructure at Leeds. The views expressed are those of the authors and not necessarily those of the NHS, the NIHR, or the Department of Health. The NIHR had no role in study design; collection, analysis or interpretation of data; the writing of the manuscript; or in the decision to submit for publication.

## CONFLICT OF INTEREST

LC has received consulting fees from Intercept Pharmaceuticals and Norgine, honoraria for lectures/speaking engagements for Intercept Pharmaceuticals, Norgine, Janssen and AbbVie, and has been financially supported to attend conferences by Intercept Pharmaceuticals, Norgine and AbbVie. None of these declarations are linked to the work of this study. None of the other authors have conflicts of interest to declare.

## ETHICS APPROVAL STATEMENT

Our study used secondary data which has been anonymised. It was approved by the School of Medicine Ethics Committee – University of Leeds (Ref no: MREC16‐046).

## PATIENT CONSENT STATEMENT

Respondents gave informed consent to data collection through completion and return of surveys. This data was subsequently anonymised to allow for secondary analysis by Approved Researchers upon written application.

## PERMISSION TO REPRODUCE MATERIAL FROM OTHER SOURCES

Our study is a secondary analysis of the VOICES National Survey of Bereaved People. The data was made available for this purpose from the UK Data Service after written application.

## Data Availability

The data that support the findings of this study are available from the UK Data Service to researchers upon application.
